# Detection of circulating tumor cells in drainage venous blood from colorectal cancer patients using a new filtration and cytology-based automated platform

**DOI:** 10.1371/journal.pone.0212221

**Published:** 2019-02-27

**Authors:** Masayuki Tsutsuyama, Hayao Nakanishi, Mayumi Yoshimura, Taihei Oshiro, Takashi Kinoshita, Koji Komori, Yasuhiro Shimizu, Yoshiyuki Ichinosawa, Seichin Kinuta, Kentaro Wajima, Yasufumi Sakakibara, Yasushi Yatabe, Seiji Ito, Yasuhiro Kodera

**Affiliations:** 1 Department of Gastroenterological Surgery, Aichi Cancer Center Central Hospital, Chikusa-ku, Nagoya, Japan; 2 Department of Gastroenterological Surgery, Nagoya University Graduate School of Medicine, Showa-ku, Nagoya, Japan; 3 Department of Pathology and Molecular Diagnostics, Aichi Cancer Center Central Hospital, Chikusa-ku, Nagoya, Japan; 4 Laboratory of Pathology and Clinical Research, Aichi Cancer Center Aichi Hospital, Okazaki, Aichi, Japan; 5 Optnics Precision Co. Ltd., Ashikaga, Tochigi, Japan; 6 Maruyasu Industries Co. Ltd., Okazaki, Aichi, Japan; Osaka Shiritsu Daigaku, JAPAN

## Abstract

Numerous technologies exist to detect circulating tumor cells (CTCs), although reports on cytological detection of CTCs remain limited. We recently developed a cytology-based CTC detection device using glass slides and light microscopy. In this study, we automated this previously manual device to improve its efficiency and cost effectiveness for clinical applications. We conducted a pilot study using this device to compare CTCs in peripheral blood (PB) and draining venous blood (DVB) from patients with colorectal cancer (CRC). The cytology-based automated CTC detection platform consisted of a disposable filtration device with a three-dimensional (3D) metal filter and multichannel automated CTC enrichment device. This platform allowed rapid and gentle filtration of CTCs and their efficient transfer from the filter to glass slides for subsequent Papanicolaou (Pap) and immunocytochemical (ICC) staining. Cytological diagnosis of CTCs was performed by observing permanent glass slide specimens by light microscopy. The current pilot clinical study enrolled CRC patients (n = 26) with stage I–IV tumors, who underwent surgery. PB was collected before surgery, and DVB was obtained from the mesenteric vein immediately after resection. Based on the CTC morphology obtained from PB and DVB samples, we proposed the following cytological criteria for the diagnosis of CTCs: pan-cytokeratin-positive, atypical cells with malignant morphological features identified by Pap staining. The numbers of CTCs defined by these criteria were significantly higher in DVB than PB from CRC patients (*p*<0.01), and the number of CTCs in DVB was increased significantly with stage progression (*p*<0.05). These results suggest that DVB may be another potential source of CTCs other than PB for liquid biopsies including downstream analysis. This automated cytology-based CTC detection device therefore provides a unique and powerful tool to investigate the significance of CTCs in CRC patients in a clinical setting.

## Introduction

Colorectal cancer (CRC)-related mortality has decreased over the past two decades as a result of advances in treatment modalities such as pre- and post-operative chemotherapies in combination with antibody-based therapies. However, mortality due to metastatic CRC has remained essentially unchanged in the past decade and is still the most common cause of CRC-related death [1]. Conventional diagnostic techniques, such as serum tumor markers and computed tomography/positron emission tomography, are usually ineffective for early diagnosis of metastasis and to evaluate the risk of postoperative metastatic recurrence in CRC patients [2]. Although cell-free circulating tumor DNA has recently emerged as a new form of liquid biopsy to detect genetic alterations in patients with advanced stage CRC, its use as an early diagnostic marker for metastasis is still limited [3].

Circulating tumor cells (CTCs) have long been a major candidate marker for liquid biopsy, and they remain a major focus of basic and clinical studies of metastasis. Many recent studies have reported the significance of CTCs as a prognostic and diagnostic marker in CRC [4, 5]. CTC numbers before treatment are a good indicator of survival in patients with metastatic disease, and CTCs are also a potentially useful predictor of relapse after surgery and combined modality therapy [6]. However, despite accumulating evidence for the application of CTCs in CRC, their significance in routine clinical practice remains limited. This is partly because the numbers of CTCs detected in peripheral blood (PB) are sometimes too small and their incidence too low for reliable and routine clinical applications such as immunocytochemical (ICC) and genetic analyses [7]. In addition, methods to detect CTCs vary greatly with few gold standard detection methods except for the CellSearch system [8]. To date, CTCs have usually been identified by immunophenotypical criteria such as keratin+/EpCAM+/CD45−/DAPI+ as judged by dark field immunofluorescence (IF) [9]. However, this approach cannot provide sufficient morphological and cytological information on CTCs and their background cellular constituents in blood. Furthermore, CTC detection platforms based on epitope-dependent IF criteria have been associated with the risk of missing CTCs with the epithelial-mesenchymal transition (EMT) phenotype [10].

In contrast, cytology-based CTC detection methods are independent of epitopes, thereby potentially reducing the incidence of false negative results. There are currently several cytology-based CTC detection platforms employing light microscopy, but few of these employ CTC detection devices that use CTC-attached glass slides, which allows subsequent cytological examination by conventional Papanicolaou (Pap) and ICC staining [11]. We recently developed a filtration-based microfluidic CTC detection device employing a unique three-dimensional (3D) metal filter [12]. This 3D filter enables efficient filtration of CTCs and gentle transfer of CTCs from the filter to a glass slide with minimum stress on the CTCs, allowing cytological staining of CTC slides by Pap and cytokeratin ICC [13]. However, this device had a slow flow rate and was operated manually with one specimen analyzed at a time, resulting in low cost effectiveness and making it unsuitable for clinical application. In the present study, we aimed to overcome these limitations by developing an improved, disposable, and automated CTC detection platform with high cost performance. In addition, to overcome the problem of CTC, we collected CTCs from draining venous blood (DVB) that reportedly contains more CTCs than PB [14]. We conducted a pilot study using this new CTC detection platform and potentially CTC-rich source to propose cytological criteria for CTCs and examine the feasibility of these criteria for detection of CTCs in CRC patients.

## Materials and methods

### Reagents

A mouse monoclonal antibody against human wide spectrum (pan)-cytokeratin (Clone, Oscar) was purchased from BioLegend (Dedham, MA, USA). Mouse monoclonal antibodies against human CD45, CD61, CD68, and CD34 were purchased from Dako (Carpinteria, CA, USA). A Zenon Alexa Fluor 488 (568) mouse labeling kit (Invitrogen, Eugene, OR, USA) was used for direct labeling of antibodies. Hoechst 33342 (Molecular Probes, Eugene, OR, USA) was used for nuclear staining.

### Patients

Patients with stage I–IV primary CRC (n = 26), who underwent surgery without preoperative drug therapy at Aichi Cancer Center Central Hospital during 2017–18, were enrolled in this study. The average age of the patients was 66 years and the male/female ratio was 18/8. PB was collected from a cubital vein into EDTA-2K tubes before surgery, and DVB was obtained from the main trunk of the mesenteric vein of the resected intestine using an 18 G needle within several minutes after resection. PB from healthy volunteers (n = 14) was used as a negative control. Healthy volunteers showed characteristics such as median age (range) of 55 (35–64) and gender ratio (male/female) of 10/4. We confirmed healthy controls as non-cancerous volunteers based on the results of periodic medical examination. The blood samples were kept at room temperature and used for examination within 2 h. The tumors ranged from stage I to IV, and their histology was mostly moderately differentiated adenocarcinoma based on the Union for International Cancer Control criteria (Table 1). This study was approved by the institutional ethics review board of the Aichi Cancer Center (Approval number: 2016-1-359), and written informed consent was obtained from each patient prior to sample collection. The study met the standards defined by the principles outlined in the Declaration of Helsinki.

**Table 1:** Patient’s characteristics.

Parameters		n = 26	Median (range)
Age			66 (39–80)
Gender	Male	18	
	Female	8	
Location	Right side colon	6	
	Left side colon	4	
	Rectum	16	
Tumor size (mm)			33 (1–150)
Histology	Differentiated	24	
	Poorly differentiated	2	
pStage	I	7	
	II	6	
	III	7	
	IV	6	
Distant metastasis	Liver	4	
	Lymph node	2	

### Cell lines

Cell-spiking experiments were carried out using COLM-5 human colon cancer cells established in our laboratory [15]. The cells were maintained in Dulbecco’s modified Eagle’s medium (Nissui Co., Tokyo, Japan) containing 10% fetal bovine serum (Gibco, Grand Island, NY, USA) penicillin, and streptomycin sulfate (Sigma-Aldrich, St. Louis, MO, USA) at 37°C in a humidified atmosphere with 5% CO_2_.

### CTC detection

**Fig 1:**
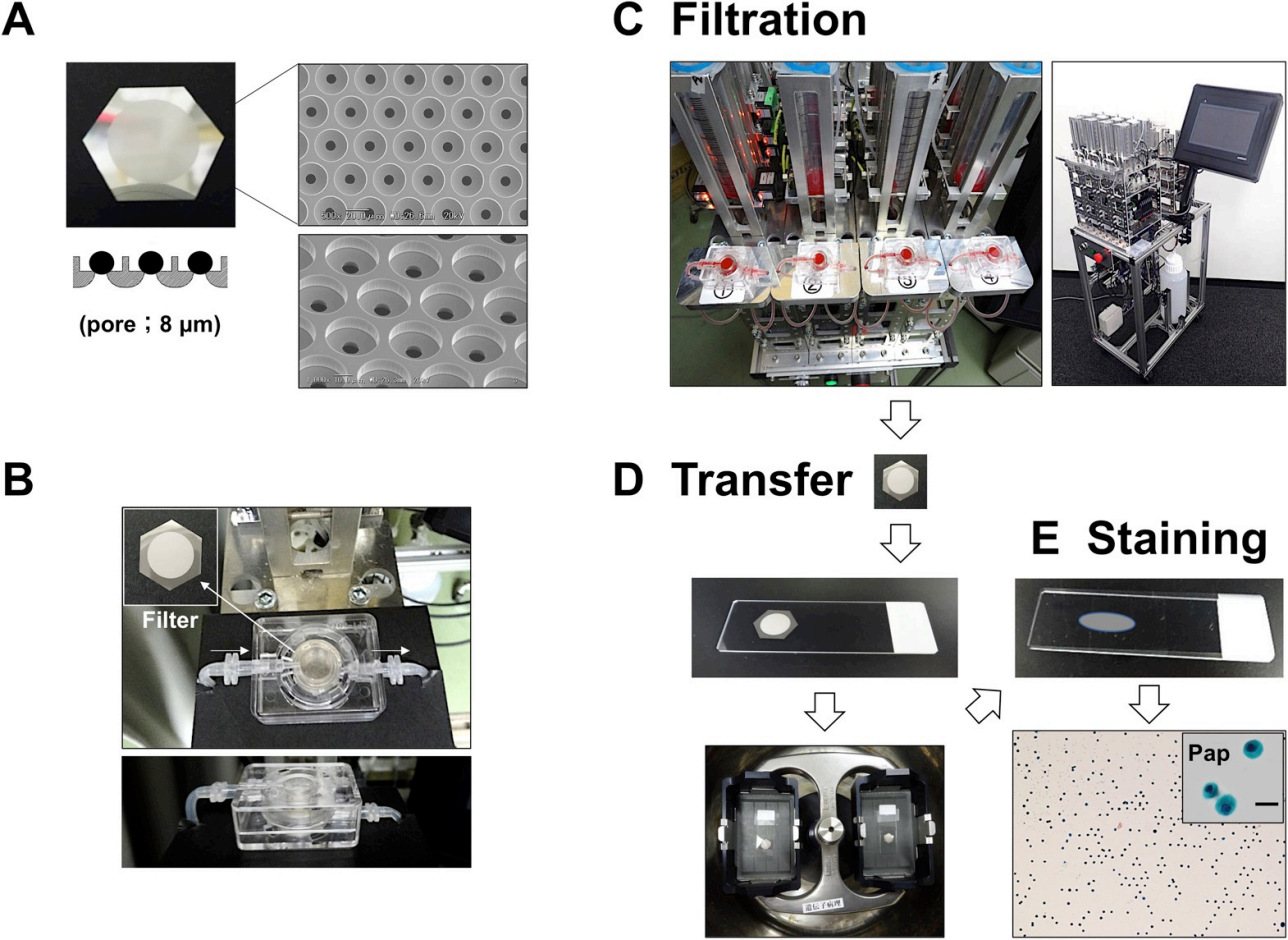
Flowchart of the CTC detection method using an automated cytology-based, microfluidic platform. **(**A) Macroscopic view and scanning electron microscope image of the 3D metal (nickel) filter. (B) Overview of the disposable CTC filtration device containing a 3D filter inside. (C–E) Overview of sequential processes of CTC detection including filtration, CTC transfer to a glass slide, and staining. (C) Appearance of the multi (four)-channel automated CTC enrichment apparatus with a monitor panel. (D) Transfer of tumor cells (COLM-5) from the 3D filter to a glass slide by brief centrifugation. (E) Resultant transferred tumor cells on a glass slide after centrifugation visualized by Pap staining (right column). Inset: enlarged view of Pap-stained tumor cells. Bar = 20 μm.

CTCs were detected as follows: 1) enrichment of CTCs using a disposable 3D filter device with an automated liquid delivery apparatus (Fig 1A–1C). 2) transfer of CTCs from the filter to a glass slide by brief centrifugation (Fig 1D). 3) subsequent cytological staining of CTCs on glass slides by Pap and ICC staining on separate slides or on the same slide (Fig 1E).

### Fabrication of a metal filter and disposable filtration-based microfluidic device

The filtration-based microfluidic device containing a 3D filter in the center was previously produced using a 3D printer [13]. To improve the durability and cost of the device, we developed a new device by injection-molding technology using a polycarbonate polymer with high durability and low cost, allowing the creation of a disposable, cost-effective device (Fig 1B) (Optnics Precision Co., Ltd., Tochigi, Japan). The CTC filtration device employed a 3D metal (pure nickel or nickel alloy) filter with 8 μm pores in the lower layer, and a CTC capture hole (10 μm height x30 μm diameter) in the upper layer (Fig 1A). This 3D filter was produced by microfabrication technology, involving X-ray lithography and electroforming processes (Optnics Precision Co.), as described previously [12].

### Filtration and enrichment of CTCs by microfluidic device

**Fig 2:**
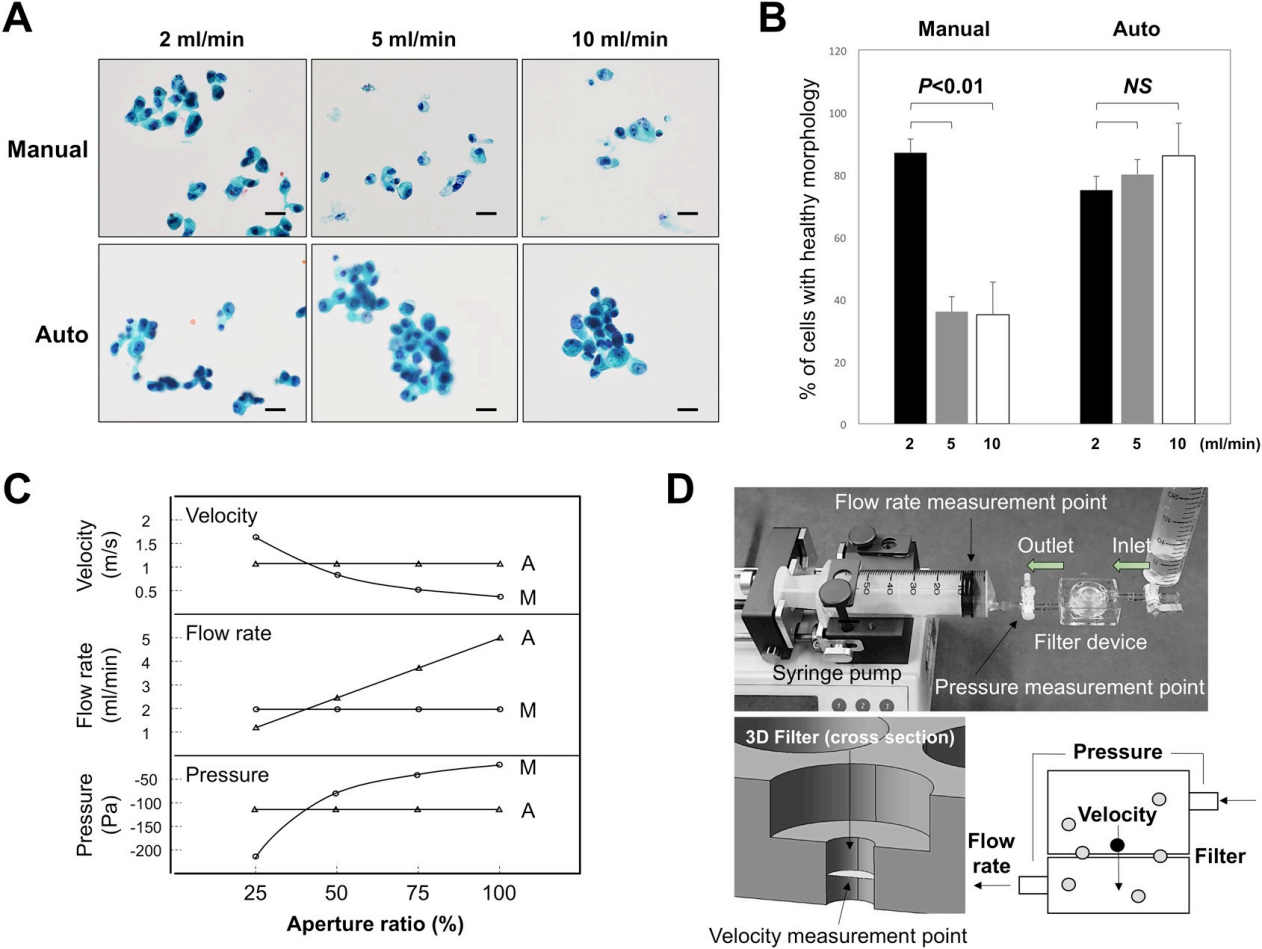
Comparison of the flow rate and cell morphology between manual and automated filtration apparatuses. (A) Effect of flow rate on the morphology of COLM-5 tumor cells transferred to glass slides and stained with Pap. (B) Changes in the percentage of tumor cells with healthy morphology transferred to glass slides with increasing flow rate. *P*<0.01 (2 ml/min vs 5, 10 ml/min). NS: not significant. Bars = standard deviation (SD). (C) Changes in parameters such as pressure, flow rate, and velocity at each measurement point with a decreasing aperture ratio. Comparison of the manual device with a syringe pump and the automated apparatus with a pressure control system. A = automated, M = manual. (D) Overview of the manual-type microfluidic device using a syringe pump as a control to compare with the automated device. Three measurement points are shown. Schematic representations of each parameter are also shown in the right lower. Gray circles = leukocytes, Black circle = CTC.

Patient whole blood (5–10 ml for PB and 2–16 ml for DVB) was diluted 10-fold with phosphate-buffered saline (PBS) containing 5 mM EDTA. This diluted blood was filtered and washed with PBS containing 5 mM EDTA using a disposable filtration device (Optnics Precision Co., Ltd.) (Fig 1A and 1B) at a flow rate of 5–10 ml/min using an automated liquid delivery apparatus (Fig 1C). For comparison, the previous, manual-type filtration platform using a syringe pump was also used for CTC enrichment (Fig 2D), as described previously [13].

### Development of an automated CTC enrichment apparatus

The automated CTC enrichment apparatus with a fluid pressure control system consisted of a diaphragm pump (Nidec, Kyoto, Japan), negative pressure regulator (SMC, Tokyo, Japan), electromagnetic valve, and liquid level sensors (Panasonic, Osaka, Japan) regulated by a programmable logic controller (Omron, Kyoto, Japan). A key point for the fluid pressure control system in this automated system is the use of a negative pressure regulator containing a pressure sensor and electromagnetic valve in combination with the diaphragm pump which allows stable supply of negative pressure. This automated apparatus included a multichannel device able to run at least four blood samples on four microfluidic devices simultaneously (Maruyasu Industries Co., Ltd., Okazaki, Japan) (Fig 1C).

After automated filtration of the whole blood samples, CTCs on the filter were fixed in the device with 10% buffered formalin for 30 min followed by washing with PBS containing 5 mM EDTA. The 3D metal filter was then detached from the microfluidic device and placed upside down on a coated glass slide (MAS coat, Matsunami, Osaka, Japan) and immersed in 100 μl PBS/EDTA with a glass cover slip. The CTCs on the filter were then quickly transferred to the glass slide by brief centrifugation (500 *g* for 10–20 sec) using a swing rotor (T5S32) with the highest acceleration rate at room temperature (Hitachi Himac CF16RX, Tokyo, Japan) (Fig 1D). The recovery rate of CTCs from filter to a glass slide was at least more than 50%. The resultant glass slide with the attached CTCs (CTC glass slide) was immediately immersed in 95% ethanol (≥60 min) for subsequent Pap staining or 95% ethanol followed by 10% buffered formalin (for 20 min) for ICC. The coverslip and filter separated spontaneously from the glass slide in the solutions under their own weight, making the CTC glass slide ready to use for cytological staining (Fig 1E).

### Cytology and ICC using CTC glass slide specimens

Conventional Pap staining of CTC glass slides was carried out using an automatic stainer (Sakura Fintec, Tokyo, Japan). For ICC, after blocking endogenous peroxidase with 0.3% H_2_O_2_ and nonspecific reactions with 1% bovine serum albumin, the CTC glass slides were incubated with a mouse monoclonal antibody against human pan-cytokeratin (or monoclonal antibodies against human CD34, CD45, CD61, or CD68) at optimal dilutions for 1–2 h. After washing, the specimens were incubated with horseradish peroxidase-labeled polymer conjugated with a goat anti-mouse antibody (EnVision+/HRP, Dako) for 30 min and then washed with PBS. The chromogen was then developed using the Liquid DAB+substrate chromogen system (Dako). Nuclei were counterstained with Meyer’s hematoxylin. Alternatively, immunostaining of CTC glass slides was carried out using the EnVision FLEX+ system with Dako Autostainer Link48 (Agilent Technologies, Santa Clara, CA, USA), according to the manufacturer’s protocol with some modifications. For combined Pap and ICC staining, Pap-stained slides were de-stained in descending concentrations of ethanol, followed by ICC for pan-cytokeratin using the same slide.

CTCs could usually be distinguished morphologically from leukocytes, macrophages, megakaryocytes, and endothelial cells by cytokeratin ICC plus Pap staining. In the event of difficulty distinguishing CTCs from the other cells described above, ICC of CD45 or CD68, CD61, and CD34 either alone or combined with Pap staining was carried out for confirmation. Alternatively, triple IF staining was first performed using a directly fluorescence labeled-antibody cocktail, e.g., an Alexa Fluor 488-conjugated mouse anti-human pan-cytokeratin antibody and Alexa Fluor 568-conjugated mouse anti-human CD45 antibody with counterstaining by Hoechst 33342, followed by fluorescence imaging, and then subjected to Pap staining. CTC specimens stained with Pap/ICC were observed and photographed under a light microscope (BX50, Olympus, Tokyo) with a CCD camera and/or observed under an inverted fluorescence microscope (Eclipse Ti-S, Nikon, Tokyo, Japan).

### Statistical analysis

The significance of differences between groups was determined by the Student’s *t*-test and Wilcoxon’s signed rank test. A *p*-value of less than 0.05 was considered as significant.

## Results

### Enrichment and detection of CTCs using a filtration-based automated device

CTC detection procedures included three steps, as described in the Materials and Methods (Fig 1C–1E). In the first CTC enrichment step, the previous method used a manual device with a syringe pump with no fluid pressure control system (Fig 2D). The manual device required a maximal flow rate of 2–3 ml/min to maintain healthy tumor cell (COLM-5) morphology with cells becoming unhealthy at higher flow rates (5–10 ml/min) (Fig 2A, upper column). Morphology of tumor cells, either healthy or unhealthy, was estimated by Pap staining based on morphological criteria such as intact cytoplasm, clear nucleoli, and the chromatin pattern. In contrast, the new automated liquid delivery apparatus with the pressure control system maintaining constant pressure allowed a higher flow rate (5–10 ml/min) and intact morphology of the captured cells on a glass slide (Fig 2A, lower column). Quantitative analysis showed that the percentage of cells with healthy morphology was significantly decreased with an increasing flow rate in the manual device (*p*<0.01), but unchanged with the increasing flow rate in the automated device (Fig 2B), indicating that the automated system with the fluid pressure control system allowed 2–4 times more rapid filtration of CTCs with healthy morphology than the manual-type device.

**Fig 3:**
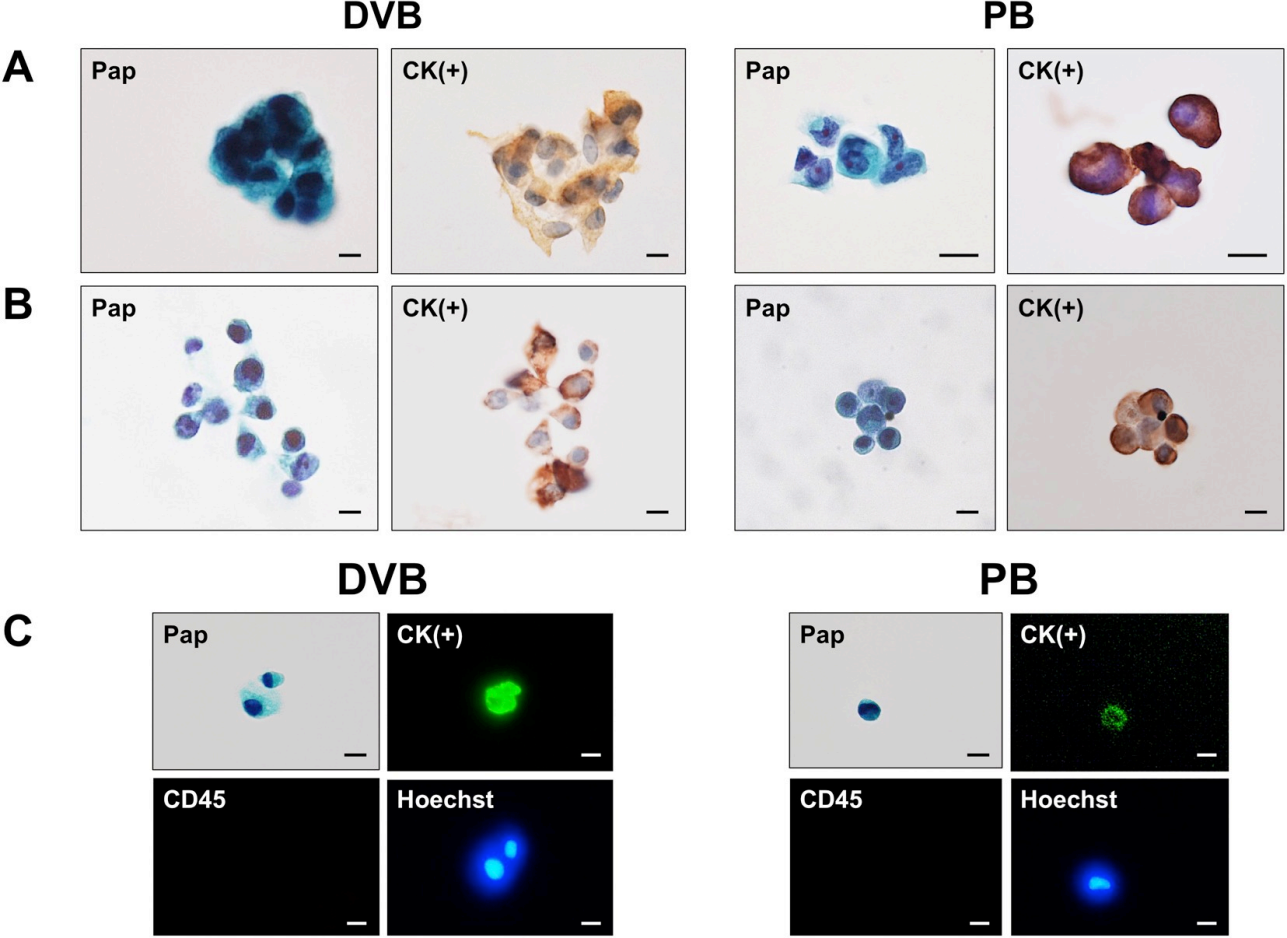
Identification of CTCs in peripheral blood (PB) and draining venous blood (DVB) from CRC patients. (A) Representative CTCs in DVB and PB from the same patient detected by pan-cytokeratin ICC and Pap staining with separate specimens. (B) Representative CTCs in DVB and PB stained by combined Pap and pan-cytokeratin ICC on the same specimen. De-stained Pap slide used for subsequent ICC. (C) Representative CTCs in DVB and PB stained by combined Pap and triple IF (Alexa Fluor 488-cytokeratin/Alexa Fluor 568-CD45/Hoechst 33342) on the same specimen. Bar = 10 μm.

We investigated the reason for the differences in CTC morphology between cells filtered using manual and automated devices by measuring three parameters such as pressure, flow rate and velocity of the cells using respective sensors. In the manual device using a syringe pump with a constant flow rate (Fig 2D), the negative pressure and velocity were increased with the decrease in aperture ratio of the filter (number of 8 μm pores occupied by blood cells/total pore number of a filter) (Figs 2C and 3M), whereas in the automated device with a pressure control system (Fig 1C), the pressure and velocity were unchanged with the decrease in aperture ratio of the filter (Fig 2A and 2C), maintaining the healthy morphology of cells even at a rapid flow rate probably as a result of the low shear stress on the cells during capture in the filter pores of the automated device (Fig 2A).

### Cytological criteria for CTC identification

**Fig 4:**
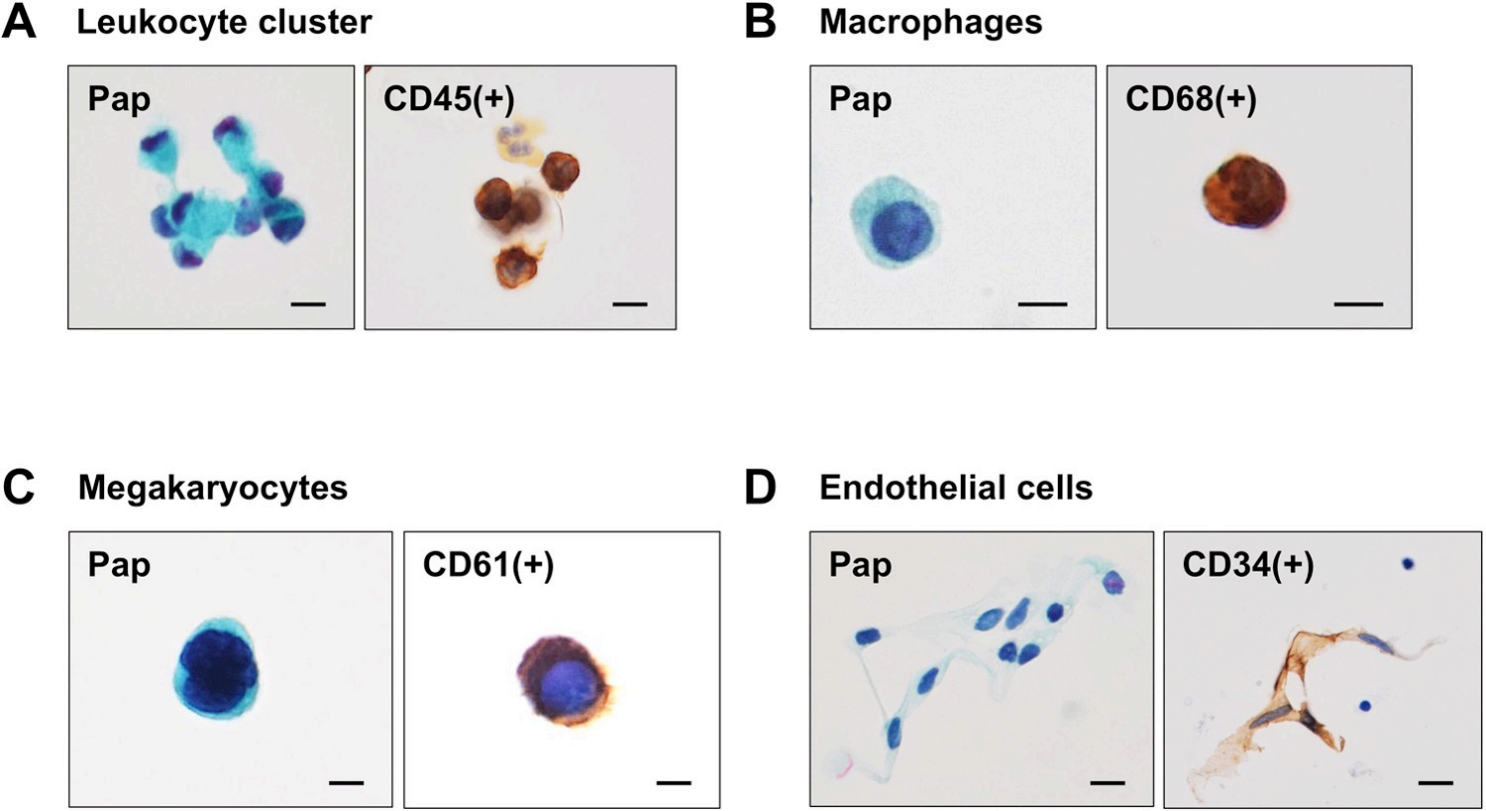
Differentiation of CTCs from circulating non-neoplastic cells in DVB and PB from CRC patients. (A) Leukocyte cluster stained for Pap/CD45 and a macrophage stained for Pap/CD68. (B) Circulating megakaryocytes in PB stained for Pap/CD61, showing multinucleated giant cell morphology. (C) Circulating endothelial cells in DVB stained for Pap/CD34, showing cell cluster formation. Bar = 10 μm.

For routine measurement of CTCs, each patient blood sample was divided into two tubes, and two CTC slide specimens were prepared after filtration: one specimen was used for pan-cytokeratin ICC with hematoxylin counterstaining and the other for Pap staining to assist with correct morphological identification of CTCs (Fig 3A). Alternatively, a CTC slide first stained with Pap could be de-stained, followed by ICC for pan-cytokeratin using the same slide (Fig 3B), or followed by triple IF staining for pan-cytokeratin, CD45, and Hoechst 33342. Triple IF staining, followed by Pap was also possible (Fig 3C). Based on these results, we identified CTCs as pan-cytokeratin-positive cells with atypical morphology supported by Pap staining. Other cells sometimes encountered in PB and DVB, which might potentially be confused with CTCs morphologically included circulating megakaryocytes, endothelial cells, enlarged macrophages, and leukocyte clusters, but these cells were usually distinguished from CTCs morphologically after Pap staining (Fig 4A–4C). The most critical candidate cells requiring careful identification were circulating endothelial cells, because of their morphological similarity to CTCs (e.g. cluster formation) and relatively high incidence, especially in DVB (Fig 4D). Atypical cells difficult to distinguish from candidate cells by Pap staining and cytokeratin ICC could be confirmed by triple IF for CD34 (endothelial cells), CD61 (megakaryocytes), CD68 (macrophages), or CD45 (leukocytes) using the same specimens (Fig 3C). Based on the various staining results, we considered that CTCs were defined cytologically as pan-cytokeratin-positive atypical cells with malignant morphological features, excluding endothelial cells, megakaryocytes, macrophages, and some leukocyte clusters.

### Detection of CTCs in PB and DVB from CRC patients

**Fig 5:**
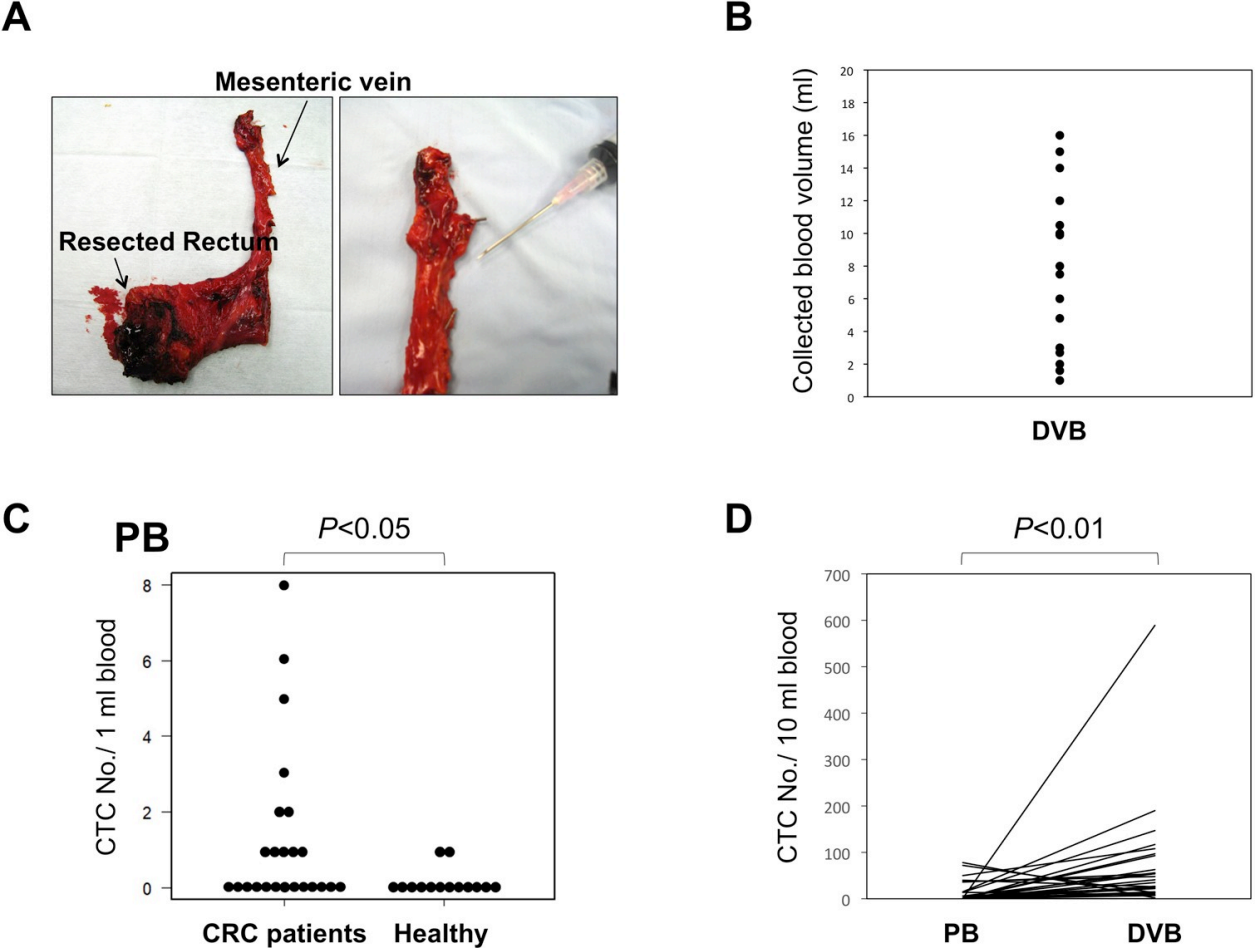
Collection of DVB and numbers of CTCs in DVB and PB from CRC patients. (A) DVB was obtained by puncture of the main trunk of the mesenteric vein of the resected colon. Enlarged view of mesenteric vein (right). (B) Variations in DVB volumes collected. (C) Numbers of CTCs in PB from CRC patients (n = 26) and healthy volunteers (n = 14). *p*<0.05. (D) Numbers of CTCs in PB and DVB from individual CRC patients (n = 26). *p*<0.01.

**Fig 6:**
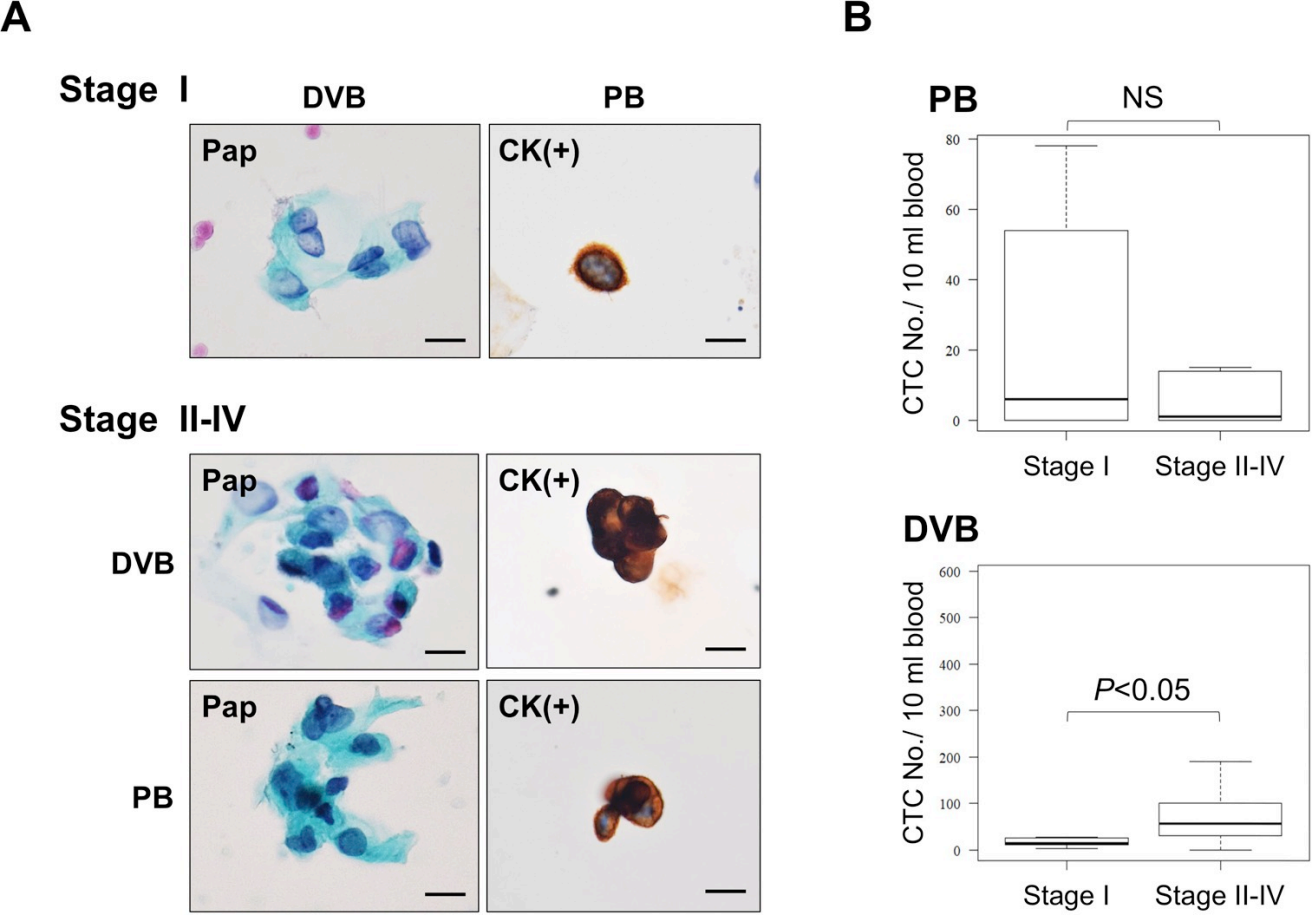
Numbers of CTCs in PB and DVB from CRC patients in relation to the tumor stage (I–IV). (A) Representative CTCs stained by Pap and cytokeratin ICC from patients with stage I (upper) and stage II–IV CRC (lower). Bar = 10 μm. (B) Numbers of CTCs in PB and DVB from CRC patients according to stage. *p*<0.05 (Stage I vs Stage II–IV) for DVB. NS (= not significant) for PB. Bars = standard deviation (SD).

Using this CTC enrichment platform and cytological criteria for CTCs, we evaluated the feasibility of the CTC detection system using PB and DVB from 26 CRC patients (Table 1) and PB from 14 healthy volunteers. DVB ranging from 2 to 16 ml was successfully obtained from the mesenteric vein of almost all patients (Fig 5A and 5B). CTC-like cells with atypical morphology were extremely rare in PB of the healthy volunteers, except for rare megakaryocytes. The number of CTCs in PB was significantly higher in CRC patients than in healthy volunteers (*p*<0.05) (Fig 5C). The CTC positivity rates (≥1) for PB and DVB from CRC patients were 54% and 96%, respectively, and the numbers of CTCs ranged from 0–78 and 0–589 per 10 ml blood in PB and DVB, respectively, indicating the presence of significantly more CTCs in DVB compared with PB (*p*<0.01) (Fig 5D). Furthermore, we analyzed the correlation between the CTC number and tumor stage, and found that the number of CTCs tended to increase with stage progression (stage I versus stage II–IV) in DVB (*p*<0.05), but not in PB (Fig 6A and 6B), indicating that the CTC number in DVB may be a potentially useful diagnostic and prognostic indicator for CRC patients.

## Discussion

In the present study, we improved on our previous device [13] and developed a new automated cytology-based, multichannel CTC detection platform with several unique characteristics. The new device is a disposable filtration device with low cost produced by injection molding technology, containing a 3D nickel filter to detect CTCs with high cost efficiency, making it suitable for clinical applications. The inclusion of an automated enrichment apparatus with a constant negative pressure allowed 2–4 times more rapid filtration of CTCs with healthy morphology than the previous manual device at a constant flow rate. Furthermore, Pap staining in combination with ICC (or IF) for pan-cytokeratin or other CD antigens using CTC glass slides improved the accuracy of cytological detection of CTC by light microscopy.

Several studies have previously reported filtration-based cytopathological detection methods for CTCs [16–18]. Hofman *et al*. conducted a blinded, multicenter study using a blood filtration method with polycarbonate membrane filters, followed by direct membrane staining by the Giemsa method. They showed that conventional cytopathological detection of CTCs based on morphological criteria, such as circulating non-hematological cells with malignant features, was a promising approach to detect CTCs in clinical oncology [19]. More recently, Adam *et al*. isolated CTCs using a precision microfilter produced by lithographic technology (Cellsieve). After filtration, CTCs on the filter were directly stained using an antibody cocktail against cytokeratin/EpCAM/CD45, followed by further staining with hematoxylin and eosin for cytopathological estimation [20, 21]. However, detection of CTCs in many of these previous filtration-based methods was dependent on direct staining of CTC-captured filters, which has some drawbacks in terms of microscopic analysis, including the fact that the thin polymer membrane is microscopically not always flat, and the filter pores or leukocytes and their debris retained in the pores negatively affect the microscopic observation of CTCs under bright or dark fields. In this respect, our current cytology-based platform has some advantages, including the transfer of CTCs to a flat glass slide with no pores, making it suitable for Pap staining similar to conventional exfoliative cytology. The new platform using CTC glass slides also permits a variety of combination staining options for Pap/ICC and Pap/triple IF (or triple IF/Pap), allowing reliable identification and further characterization of CTCs. Furthermore, the platform can detect CTCs with an EMT phenotype by Pap staining-assisted ICC using a pan-cytokeratin antibody that even stains undifferentiated carcinoma cells [22, 23]. The current CTC detection system is based on permanent specimens and can thus be available in a clinical laboratory, allowing more objective judgment of CTCs by multiple cytopathologists by light microscopy. In this respect, Xu *et al*. recently reported a similar size-based (not filter-based) microfluidic platform able to visualize CTCs transferred from a cassette to glass slides by IF staining (Parsortix) [24]. However, their staining system depended on IF rather than Pap and ICC staining. To the best of our knowledge, the current CTC detection device reported in this study is the first filter-based platform for cytological (Pap staining) and immunocytological detection of CTCs using permanent glass slides observed by light microscopy.

To evaluate the current cytology-based platform in a clinical setting, we examined CTCs in PB and DVB samples from patients with primary CRC. Although this was a pilot study with a small sample size (n = 26), the results obtained from the clinical blood samples suggested that CTCs could be defined by the following immunocytological criteria assisted by Pap staining: pan-cytokeratin-positive, atypical cells with malignant morphological features, excluding leukocyte clusters, large macrophages, circulating megakaryocytes, and endothelial cells. Based on these cytological criteria, we examined blood samples from CRC patients and demonstrated that CTCs were significantly increased in DVB compared with PB from CRC patients (*p*<0.01). Furthermore, the number of CTCs in DVB, but not PB, tended to increase with progression from early to advanced tumor stage (stage I vs stage II–IV) (*p*<0.05). These results are consistent with previous reports [25, 26]. Considering that DVB can be sufficiently collected from patients after surgery without risk, CTCs in DVB may provide a potentially useful CTC-rich source as a liquid biopsy in CRC patients.

The current cytology-based CTC filtration device has some limitations and is still in the development process in terms of mainly following two points; 1) Relatively low efficiency of transferring CTCs from filter to a glass slide. Recovery rate of CTCs to glass slide by centrifugation method used in this study is relatively low ranging from 50% to 70%. 2) Morphology of CTCs on the glass slide is not always best-preserved and the distribution of CTCs is somewhat heterogenous on the glass slide. These two weakness with our present CTC detection device are largely due to the centrifugation method for CTC transfer. To overcome these problems, we are now developing a new CTC transferring method using a pressure-type device via either air pressure or direct pushing force rather than centrifugal force. Further study is needed for optimization of this method.

In conclusion, we developed a new automated cytology-based, cost-effective CTC detection platform and defined cytological criteria for CTC identification based on light microscopic observation of immunocytologically stained specimens. This cytology-based CTC detection platform, combined with the use of DVB as a new CTC source, offers a potentially useful prognostic and diagnostic tool as a liquid biopsy in patients with metastatic CRC. A further large scale clinical study is ongoing to clarify the clinical significance of CTCs in DVB from patients with CRC at our institute.
